# *In Situ* Capture RT-qPCR: A New Simple and Sensitive Method to Detect Human Norovirus in Oysters

**DOI:** 10.3389/fmicb.2017.00554

**Published:** 2017-04-03

**Authors:** Zhenhuan Zhou, Zhengan Tian, Qianqian Li, Peng Tian, Qingping Wu, Dapeng Wang, Xianming Shi

**Affiliations:** ^1^MOST-USDA Joint Research Center for Food Safety, School of Agriculture and Biology, Shanghai Jiao Tong UniversityShanghai, China; ^2^State Key Laboratory of Applied Microbiology Southern China, Guangdong Provincial Key Laboratory of Microbial Culture Collection and Application, Guangdong Open Laboratory of Applied Microbiology, Guangdong Institute of MicrobiologyGuangzhou, China; ^3^Shanghai Entry-Exit Inspection and Quarantine Bureau of P.R.CShanghai, China; ^4^Department of Bioengineering, Shanghai Institute of TechnologyShanghai, China; ^5^Produce Safety and Microbiology Research Unit, Western Regional Research Center, Agricultural Research Service, United States Department of AgricultureAlbany, CA, USA

**Keywords:** human noroviruses, *in situ* capture RT-qPCR, clinical sample, oyster

## Abstract

Human noroviruses (HuNoVs) are the major cause worldwide for non-bacterial acute gastroenteritis. In this study, we applied a novel viral receptor mediated *in situ* capture RT-qPCR (ISC-RT-qPCR) to detect HuNoVs in oysters and compared with the traditional RT-qPCR method. Ten HuNoVs RT-PCR positive and 5 negative clinical samples from gastroenteritis patients were used to compare specificity and sensitivity of ISC-RT-qPCR against that of the RT-qPCR assay. ISC-RT-qPCR had at a one-log and a two-log increase in sensitivity over that of the RT-qPCR assay for genotype I (GI) and GII, respectively. Distributions of HuNoVs in oyster tissues were investigated in artificially inoculated oysters. GI HuNoVs could be detected in all tissues in inoculated oysters by both ISC-RT-qPCR and RT-qPCR. GII HuNoVs could only be detected in gills and digestive glands by both methods. The number of viral genomic copies (vgc) measured by ISC-RT-qPCR was comparable with RT-qPCR in the detection of GI and GII HuNoVs in inoculated oysters. Thirty-six oyster samples from local market were assayed for HuNoVs by both assays. More HuNoVs could be detected by ISC-RT-qPCR in retail oysters. The detection rates of GI HuNoVs in gills, digestive glands, and residual tissues were 33.3, 25.0, and 19.4% by ISC-RT-qPCR; and 5.6, 11.1, and 11.1% by RT-qPCR, respectively. The detection rates of GII HuNoVs in gills were 2.8% by ISC-RT-qPCR; no GII HuNoV was detected in these oysters by RT-qPCR. Overall, all results demonstrated that ISC-RT-qPCR is a promising method for detecting HuNoVs in oyster samples.

## Introduction

Noroviruses (NoVs) belong to the *Caliciviridae* family. They can be classified into six genogroups (GI to GVI). Most NoVs that infect humans belong to genogroups GI, GII, and GIV, which are called human noroviruses (HuNoVs). These viruses can be further divided into more than 40 genotypes (Glass et al., 2009; Kroneman et al., 2013; Tran et al., 2013; Eden et al., 2014; Vinjé, 2015). HuNoVs are the major cause of non-bacterial acute gastroenteritis worldwide (Lopman et al., 2002; Yu et al., 2015). The virus is highly infectious, and the probability of infection by a single HuNoV virion is close to 0.5, exceeding that has been reported for any other virus studied to date (Teunis et al., 2008). It was commonly reported that HuNoVs cannot be cultivated *in vitro*, until recently a new cell culture system of HuNoVs was developed (Ettayebi et al., 2016). So far, RT-PCR and quantitative RT-PCR (RT-qPCR) have been widely used for the detection of HuNoVs (Kageyama et al., 2003; Trujillo et al., 2006; Yu et al., 2015). However, these molecular approaches have limited value in distinguishing infectious viruses from non-infectious viruses or free viral RNA (Li et al., 2014; Wang and Tian, 2014).

Histo-blood group antigens (HBGAs) have been recognized as receptors or co-receptors for HuNoVs (Hutson et al., 2002; Marionneau et al., 2002). Previously, we demonstrated that porcine gastric mucin (PGM) contained multiple human HBGAs (type A, H1, and Lewis antigens) and could be bound by multiple strains of HuNoVs (Tian et al., 2008). PGM- or synthetic HBGAs- conjugated magnetic beads have been then utilized as a method for concentrating HuNoVs (Tian et al., 2005, 2008; Cannon and Vinjé, 2008) and to estimate the inactivation status of HuNoVs treated by high-pressure processing (HPP) or heat inactivation (Dancho et al., 2012). We further improved the method by coating hybrid binding/PCR reaction containers with PGM to sequester HBGA-binding viruses, which was then followed by *in situ* amplification of the captured viral genomes by RT-qPCR (Wang and Tian, 2014; Wang et al., 2014). The cultivable Tulane Virus (TV) was used to validate this *In Situ* Capture RT-qPCR (ISC-RT-qPCR) method (Wang et al., 2014). Our previous work indicated that this method could be used for evaluating inactivation status of TV and HuNoV caused by heat and chlorine treatments (Wang and Tian, 2014; Wang et al., 2014). However, the ISC-RT-qPCR method has not been applied toward the detection of HuNoVs in clinical and food samples.

Oysters have been recognized as one of the well-known vehicles for transmission of HuNoVs in food related outbreaks (Maalouf et al., 2010, 2011). It has been reported that HuNoVs could be bio-accumulated by oysters and persist in the oyster tissues for a long period of time (Le Guyader et al., 2006, 2012). It was reported that HuNoVs could be detected in 53 out of 507 oyster samples (10.5%) from 11 countries by RT-PCR (Cheng et al., 2005). It remains unknown if these HuNoV RT-PCR-positive oysters were infectious. In this study, the presence and distribution of HuNoVs in oyster tissues were tested by ISC-RT-qPCR and compared with that of RT-qPCR assay.

## Materials and methods

### Clinical and oyster samples

#### Clinical samples and confirmation

Fifteen clinical gastroenteritis samples were kindly provided by Dr. Zhiyong Gao at Beijing Center for Disease Control and Prevention (CDC), China. All experiments involved clinical samples were performed in a BSL-2 lab. Raw stool samples were diluted into a 1:20 suspension in phosphate-buffered saline (PBS, pH 7.2, NaCl 137.0 mmol/L, KCl 2.7 mmol/L, Na_2_HPO_4_ 10.0 mmol/L, KH_2_PO_4_ 2.0 mmol/L), clarified by low-speed centrifugation (3,000 rpm) for 5 min, and stored as viral stocks at −80°C. Each sample was measured by RT-PCR as previously reported (Schwab et al., 1995) with JV12/13 primers (Table 1). The RT-PCR products were sequenced by Genewiz Bio-Technology Co. Ltd (Suzhou, China). Subsequently, the sequence results had been submitted to GenBank. Two selected samples were used to compare the sensitivity of ISC-RT-qPCR and RT-qPCR for GI and GII HuNoVs and used for inoculation of oysters.

**Table 1:** **Primers for RT-PCR and primer-probes for RT-qPCR**.

**Genogroup**	**Primer and probe**	**Sequence 5′ → 3′**	**References**
GI and GII	F-Primer JV12	ATACCACTATGATGCAGATTA	Schwab et al., 1995
	R-Primer JV13	TCATCATCACCATAGAAAGAG	
GI	F-Primer COG1F	CGYTGGATGCGNTTYCATGA	Kageyama et al., 2003
	R-Primer COG1R	CTTAGACGCCATCATCATTYAC	
	Probe^*^RING1(a)-TP	FAM-AGATYGCGATCYCCTGTCCA-TAMRA	
	RING1(b)-TP	FAM-AGATCGCGGTCTCCTGTCCA-TAMRA	
GII	F-Primer JJV2F	CAAGAGTCA ATGTTTAGGTGGATGAG	Jothikumar et al., 2005
	R-Primer COG2R	TCGACGCCATCT TCATTCACA	
	Probe RING2-TP	FAM-TGGGAGGGCGATCGCAATCT-BHQ	

* *Mixed probes are used for the GI NoVs. Y: C and T; N: A, T, G, and C*.

#### Oyster samples

Thirty-six oyster samples were collected randomly between March 2014 and February 2015 from retail markets in Shanghai as we have previously reported (Yu et al., 2016). Briefly, oysters (*n* = 3–5) were randomly purchased from retail market A and B in shanghai every 2–3 weeks and kept at 4°C during shipment. In addition, 30 oysters were randomly collected from retail market C in Shanghai in December 2014 for the inoculation assay. All oyster samples were treated within 4 h, and detected in 24 h.

### Detection of HuNoVs by ISC-RT-qPCR and RT-qPCR

#### ISC-RT-qPCR

ISC-RT-qPCR was performed as we previously reported (Wang and Tian, 2014; Wang et al., 2014). Type III PGM was purchased from Sigma (St. Louis, MI; cat. no. M-1778). Each well of hybrid binding/PCR reaction containers (Nunc Immuno Module, VWR, Brisbane, CA) was coated with 100.0 μL of PGM solutions with concentration consisting of 1.0 mg/mL in 0.05 mol/L carbonate-bicarbonate buffer (pH 9.6) at 4°C overnight. After being washed 3 times by PBS, the wells were blocked with 120.0 μL of 1.0% bovine serum albumin (BSA) in PBS at 37°C for 1 h. The wells were washed with PBS and used immediately. The sample (100.0 μL) was added into each PGM-coated well, and incubated at 37°C for 30 min. After incubation, each well was washed with PBS for 3 times. After 8.5 μL of RNase-free double distilled water (ddH_2_O) was added to each well, the binding/PCR reaction containers were sealed with polyolefin sealing tape (VWR, West Chester, PA, USA) and heated at 95°C for 5 min, followed by cooling at 4°C. ISC-RT-qPCR was performed on a qPCR system (“CFX96,” Bio-Rad; CA) using a one-step RT-qPCR kit (Vazyme, Nanjing, China) in accordance with the manufacturer's protocol. All primers and probes used in this study were listed in Table 1. Each 25.0 μL reaction consisted of 12.5 μL of one-step Q probe mix (2 ×), 2.0 μL of one-step Q probe enzyme mix, 0.5 μL of each 10.0 μmol/L primers, 1.0 μL of 10.0 μmol/L probes and 8.5 μL of template RNA from the previous step. The ISC-RT-qPCR was performed using the following amplification protocol: reverse transcription reaction at 42°C for 10 min, denaturation at 95°C for 30 s; qPCR amplification for 45 cycles consisting of denaturation at 95°C for 15 s, annealing at 53°C for 15 s, and extension at 60°C for 30 s.

#### RT-qPCR followed by RNA extraction

RT-qPCR was performed with extracted viral RNA followed by reverse transcription and qPCR amplification with the same primer-probe sets used for ISC-RT-qPCR as described above. For RNA extraction procedure, RNA was extracted from 100.0 μL of the clinical and oyster samples by using an RNA extraction kit (Tiangen, Beijing, China) according to the manufacturer's protocol. The extracted RNA was air-dried and dissolved in 10.0 μL of diethyl-pyrocarbonate (DEPC) treated water prior to the reverse transcription reaction.

#### Converting *Ct* values to genomic signal

*Ct* units were converted into viral genomic copies (vgc) using a standard curve. The slope was −1.496 cycles/log 10 for GI HuNoVs with an *R*^2^ of 0.9974 and was −1.4648 cycles/log 10 for GII HuNoVs with an *R*^2^ of 0.9991 (Tian et al., 2012).

### Artificial contamination of oysters

Oysters were randomly selected and kept at 4°C during shipment. The oysters were inoculated as reported previously (Wang et al., 2008a). Briefly, after pre-feeding overnight at room temperature, viable oysters were randomly divided into two groups and were inoculated with approximate 10^5^ vgc/mL of GI.3 (3010) or GII.4 (3009) viruses. After incubating for 24 h, the oysters were processed for future use.

### Oyster sample processing

#### Dissect oyster tissues

The tissues from each oyster were divided into gills (G), digestive glands (D; including stomach, digestive diverticula) and residual tissues (O) as previous reported (Wang et al., 2008a; Suffredini et al., 2012). PBS was added to each sample (approximate 2.0 g) at a ratio of 4:1 followed by homogenization with a homogenizer (AES Chemunex, France) at 12,000 rpm for 1 min. The homogenized samples were mixed with equal amounts of glycerol (50.0%) and were stored at −80°C for future use.

#### Treatment of processed oyster samples

A 0.5 gram aliquot of each processed sample was treated as previously reported (Henshilwood et al., 2003). Briefly, the sample was mixed with 1.0 mL of proteinase K solution (0.2 mg/mL), incubated at 37°C in a shaking incubator (200 rpm) for 60 min, and then the enzyme was inactivated in a water-bath at 60°C for 15 min. The mixture was centrifuged at 12,000 × *g* for 15 min. The supernatant (~1.0 mL) was collected and prepared for detection of HuNoVs by RT-qPCR and ISC-RT-qPCR.

### Data analysis and statistics

Analysis of variance (ANOVA) and Chi-square were utilized for data analysis and differences in means were considered significant when the *p-*value was < 0.05.

## Results

### Specificity of ISC-RT-qPCR and RT-qPCR assays

The viral stocks from the clinical samples were 1:100 diluted in PBS and determined by RT-PCR using JV12/13. The RT-PCR results showed that 5 were negatives and 10 were positives for HuNoVs. All amplified productions were sequenced and submitted to GenBank. The detail information was described as follow. All clinical samples were measured by both ISC-RT-qPCR and RT-qPCR (Table 2). All 5 known negatives registered negative by both ISC-RT-qPCR and RT-qPCR. All 10 known positives registered positive by ISC-RT-qPCR, while only 8 registered positive by RT-qPCR when screened at initial 1:100 diluted viral stocks (1:2,000 dilution from raw stool samples). These two samples (sample 1028 and 3134) were further tested positive when the viral stocks were 1:10 diluted (1:200 dilution from raw stool samples) and retested. There was no significant difference in the titers (in vgc/mL in log_10_) between these two methods (Table 2, *p* > 0.05).

**Table 2:** **Detection of clinical samples by ISC-RT-qPCR and RT-qPCR**.

**Sample (NoV strain)**	**Mean ± *SD* Log_10_(vgc/mL) for ISC-RT-qPCR**	**Mean ± *SD* Log_10_(vgc/mL) for RT-qPCR**	**GenBank number**
1036	Negative	Negative	None
2051(GI.3)	7.10 (±0.12)	6.97 (±0.15)	KX611681
2052(GI.3)	7.80 (±0.21)	7.65 (±0.18)	KX611682
3010(GI.3)	8.40 (±0.07)	8.70 (±0.30)	KX426085
4151	Negative	Negative	None
1021	Negative	Negative	None
1028(GII.4)	7.81 (±0.10)	^*^5.98 (±0.12)	KX611683
2021	Negative	Negative	None
3009(GII.4)	8.45 (±0.13)	9.02 (±0.23)	KX426082
3014(GII.Pe)	7.63 (±0.06)	7.11 (±0.04)	KX426079
3035(GII.4)	8.35 (±0.04)	7.68 (±0.05)	KX426080
3143(GII.4)	7.21 (±0.11)	^*^6.03 (±0.15)	KX426081
3148	Negative	Negative	None
4135(GII.4)	7.26 (±0.22)	6.62 (±0.24)	KX426084
4156(GII.Pe)	7.11 (±0.16)	6.89 (±0.17)	KX426083

* *Retested positive at higher concentrations (1:200 dilution from raw stool samples)*.

### Sensitivity of ISC-RT-qPCR and RT-qPCR

**Figure 1:**
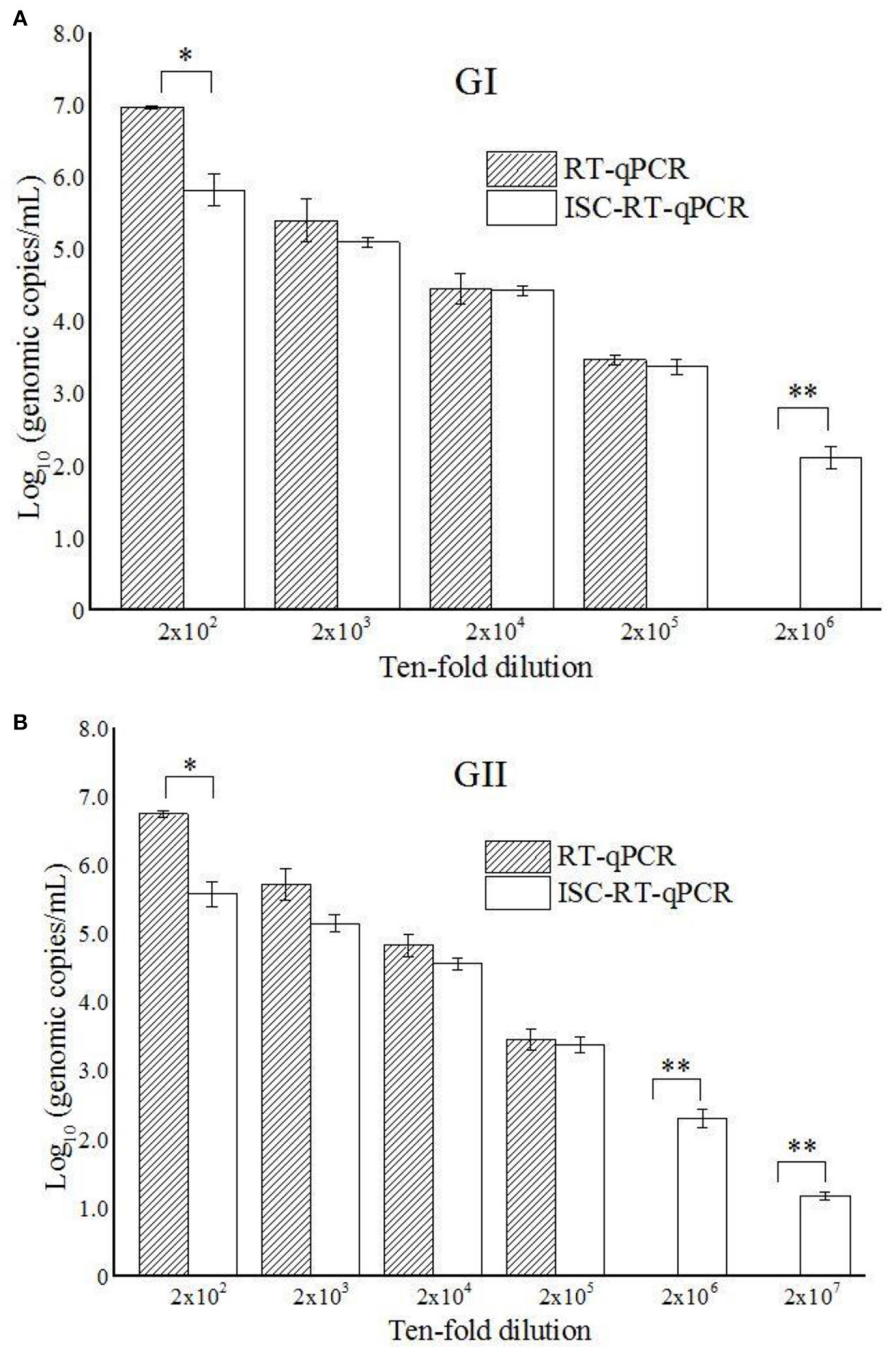
**ISC-RT-qPCR and RT-qPCR assays for GI HuNoVs (A)** and GII HuNoVs **(B)** in 10-times serial diluted clinical samples. Each data point is an average of triplicates, and each error bar represents the data range. ^*^, ^**^Represented *p* < 0.05 and *p* < 0.01 between group indicated and the rest groups.

As the prototypes of HuNoV GI and GII with known titers were not available, the sensitivity of both assays for the GI and GII HuNoVs strains were tested using clinical samples serially-diluted from 2 × 10^−2^ to 2 × 10^−7^. While RT-qPCR was able to detect both GI and GII HuNoVs strains over the dilution range of 2 × 10^−2^ to 2 × 10^−5^, the titers (in vgc/mL in log_10_) were from 6.97 (±0.02) to 3.47 (±0.07) and from 6.75 (±0.05) to 3.45 (±0.15), respectively. ISC-RT-qPCR was able to detect GI and GII HuNoVs strains over the ranges of 2 × 10^−2^ to 2 × 10^−6^, and 2 × 10^−2^ to 2 × 10^−7^, the titers (in vgc/mL in log_10_) were from 5.82 (±0.22) to 2.11 (±0.16), and from 5.58 (±0.18) to 1.17 (±0.06), respectively (Figures 1A,B). Relative to RT-qPCR, ISC-RT-qPCR exhibited a 10-fold and 100-fold greater sensitivity for extracted RNA from GI and GII HuNoVs strains, respectively.

### Detection of HuNoVs in tissues of inoculated oysters by ISC-RT-qPCR and RT-qPCR

To make sure that each oyster contained HuNoVs in their tissues, the oysters were artificially inoculated with both GI and GII HuNoVs. The distribution patterns and the titers (in vgc/mL in log_10_) of GI and GII HuNoVs in oyster tissues measured by both assays were similar (Table 3). For GI HuNoVs, the viral titers (in vgc/mL in log_10_) of G, D, and O tissues were 4.27 (±0.02), 3.87 (±0.14), and 3.63 (±0.24), measured by ISC-RT-qPCR; and were 4.12 (±0.04), 3.81 (±0.15), and 3.55 (±0.19), measured by RT-qPCR, respectively (*p* > 0.05). For GII HuNoVs, the viral titers (in vgc/mL in log_10_) of G and D tissues were also similar between the two assays. The viral titers (in vgc/mL in log_10_) were 4.11 (±0.08), 3.93 (±0.21), measured by ISC-RT-qPCR; and were 4.05 (±0.10), 3.84 (±0.18), measured by RT-qPCR (*p* > 0.05). No GII HuNoV could be detected in O tissues by either assay.

**Table 3:** **Detection of HuNoVs in artificially contaminated oyster tissues by ISC-RT-qPCR and RT-qPCR**.

**Oyster tissue**	**GI HuNoVs**		**GII HuNoVs**	
	**Mean ± *SD* Log_10_(vgc/mL) for ISC-RT-qPCR**	**Mean ± *SD* Log_10_(vgc/mL) for RT-qPCR**	**Mean ± *SD* Log_10_(vgc/mL) for ISC-RT-qPCR**	**Mean ± *SD* Log_10_(vgc/mL) for RT-qPCR**
G	4.27 (±0.02)	4.12 (±0.04)	4.11 (±0.08)	4.05 (±0.10)
D	3.87 (±0.14)	3.81 (±0.15)	3.93 (±0.21)	3.84 (±0.18)
O	3.63 (±0.24)	3.55 (±0.19)	Negative	Negative

*G represents gills, D represents digestive glands and O represents residual tissues*.

### Detection of retail oyster samples by ISC-RT-qPCR and RT-qPCR

**Figure 2:**
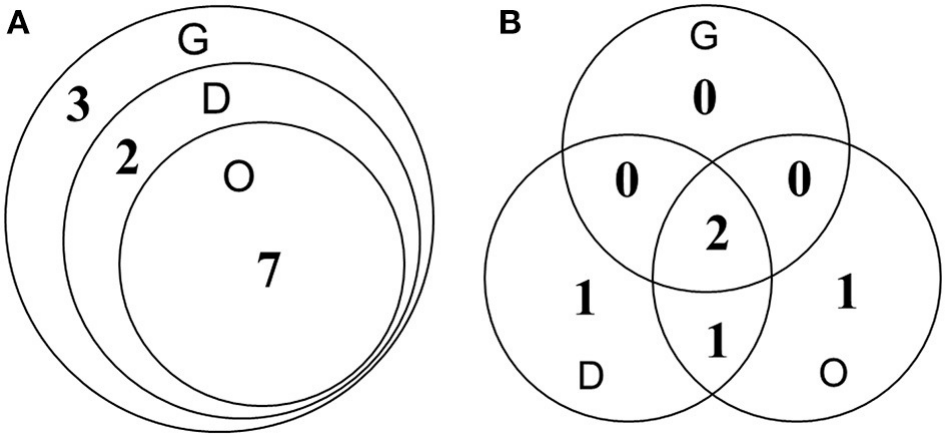
**Detection of GI HuNoVs in retail oyster samples by ISC-RT-qPCR (A)** and RT-qPCR **(B)**. G, gills; D, digestive glands; O, residual tissues. Numbers represented number of the positive samples in the overlapped area and non-overlapped area.

For the detection of HuNoVs in oysters collected from retail markets, ISC-RT-qPCR exhibited significantly better sensitivity than RT-qPCR (*p* < 0.05). Thirty-six oyster samples from retail markets in Shanghai were assayed by both methods. For detection of GI HuNoVs in tissues G, D, and O; the detection rates were 33.3, 25.0, and 19.4% by ISC-RT-qPCR, respectively; and were 5.6, 11.1, and 11.1% by RT-qPCR, respectively (Table 4A). The detection rates of GII HuNoVs in G were 2.8% by ISC-RT-qPCR; no GII HuNoV was detected in these oysters by RT-qPCR (Table 4B). For GI HuNoVs in oyster measured by ISC-RT-qPCR, whenever O tissue was positive, the corresponding D and G tissue were also positive (Figure 2A). Whenever D tissue was positive, the corresponding G tissue was also positive. Therefore, G tissue was the prefer tissue for ISC-RT-qPCR assay (Figure 2A). D and O tissues were not necessary to be tested to determine if the oyster was contaminated for ISC-RT-qPCR method. However, the target tissue for RT-qPCR assay was not obvious. For RT-qPCR, whenever G tissue was positive, the corresponding D and O tissue were also positive. On the other hand, there were some samples were only positive in D tissue or in O tissue measured by RT-qPCR (Figure 2B).

**Table 4:** **Detection of GI (A) and GII (B) HuNoVs in oyster from retail markets**.

**Oyster tissue**	**Total number**	**ISC-RT-qPCR**		**RT-qPCR**	
		**Positive number for GI**	**Positive rate for GI (%)**	**Positive number for GI**	**Positive rate for GI (%)**
**(A)**					
G	36	12	33.3	2	5.6
D	36	9	25.0	4	11.1
O	36	7	19.4	4	11.1
**Oyster tissues**	**Total number**	**Positive number for GII**	**Positive rate for GII (%)**	**Positive number for GII**	**Positive rate for GII (%)**
**(B)**					
G	36	1	2.8	0	0
D	36	0	0	0	0
O	36	0	0	0	0

*G represents gills, D represents digestive glands and O represents residual tissues*.

### Nucleotide sequence accession numbers

The amplified products from the clinical samples were sequenced and submitted to the GenBank. The GenBank accession numbers for the nucleotide sequences were as follows: 2051 (GI.3), KX611681; 2052 (GI.3), KX611682; 3010 (GI.3), KX426085; 1028 (GII.4), KX611683; 3009 (GII.4), KX426082; 3014 (GII.Pe), KX426079; 3035 (GII.4), KX426080; 3143 (GII.3), KX426081; 4135 (GII.4), KX426084; 4156 (GII.Pe), KX426083.

## Discussion

HuNoVs cause a significant public health burden worldwide (Atmar and Estes, 2006; Patel et al., 2009). RT-PCR and RT-qPCR are the most commonly-used methods for detecting HuNoVs (Fisman et al., 2009; Knight et al., 2013). Recently, RT-PCR assays have been largely replaced by the use of one-step RT-qPCR which allows for signal amplification and amplicon confirmation in a single reaction (Knight et al., 2013). RT-PCR has been mostly relegated to the sequencing and genotyping of HuNoVs. So far, many RT-qPCR primer-probe sets and protocols have been developed for the purpose of HuNoVs detection (Kageyama et al., 2003; Jothikumar et al., 2005; Trujillo et al., 2006). However, none of these methods can differentiate whether the detected signals are derived from intact (and presumably viable) virus particles, or residual RNA from degraded virus particles (Knight et al., 2013).

PGM was reported to contain type A, O and Lewis b HBGAs, which are used as receptors for the majority of HuNoVs strains (Tian et al., 2005). PGM-conjugated magnetic beads have been used to concentrate HuNoV and determine the inactivation status of HuNoV (Tian et al., 2008; Dancho et al., 2012). Recently, ISC-RT-qPCR method was also developed as an alternative method to measure the inactivation status of TV and HuNoV (Wang et al., 2014). However, the sensitivity of the PGM-based capture RT-qPCR assay has not been directly compared against that of the commonly-used RT-qPCR assay. In this study, we evaluated the specificity, sensitivity and detection limits of RT-qPCR and ISC-RT-qPCR assays using a set of clinical samples (Figures 1A,B). The specificity of the two methods tested were similar for ten RT-PCR positive samples although there were 2 samples were negative by RT-qPCR at initial screening. The two samples turned positive by RT-qPCR when re-tested at a 10-time higher concentration (1:200 dilution from raw stool samples). We further tested the detection limits of the ISC-RT-qPCR and RT-qPCR assays using serial dilutions of clinical samples (Figures 1A,B). Although the viral titers (in vgc/mL in log_10_) measured by ISC-RT-qPCR were generally lower than that of RT-qPCR between the dilution ranges of 2 × 10^2^ to 2 × 10^5^, a significant difference was only observed at the beginning of the serial dilution (1:200 dilution) probably due to an excess of viral particles beyond the binding capacity of the HBGA-coated binding/PCR reaction containers (Figures 1A,B, *p* < 0.05). In contrast, ISC-RT-qPCR was more sensitive than RT-qPCR in samples with fewer viruses. Compared to RT-qPCR, the detection limit of ISC-RT-qPCR was 10-fold lower for GI HuNoVs and 100-fold lower for GII HuNoVs. Overall, ISC-RT-qPCR exhibited a better sensitivity with low-titer viral samples. This situation was exact that the HuNoVs were in food or environmental samples.

HuNoVs outbreaks are often caused by consumption of contaminated oysters. GI HuNoVs strains have been more frequently encountered in oyster-related outbreaks, and noted to bio-accumulate in various oyster tissues (Le Guyader et al., 2012; Kittigul et al., 2016). GII HuNoVs strains have been also found in oyster tissues, but have been noted to bio-accumulate at very low levels (Wang et al., 2008b; McLeod et al., 2009; Le Guyader et al., 2012). GIV HuNoV was less common in China. The detection rate was less than 0.5% (2 out of 454 clinical samples) in stool samples tested. Therefore, detection of GIV of HuNoV was not included in this study. In this study we used both ISC-RT-qPCR and RT-qPCR assay to study the distribution of HuNoVs in various tissues of oysters. As not all retail oysters contained HuNoVs, we artificially inoculated HuNoVs to make sure each oyster was contaminated with both GI and GII HuNoVs. GI HuNoVs could be detected in all three types of tissues by both assays. However, GII HuNoVs could be detected only in G and D tissues by both assays. No GII HuNoV was detected in O tissues by both assays. We did not find significant differences in the titers (in vgc/mL in log_10_) in each tissue of HuNoV-inoculated oysters measured by ISC-RT-qPCR and RT-qPCR (*p* > 0.05). Higher vgc was observed in G tissues for both GI and GII HuNoVs than in D tissues (*p* < 0.05) and O tissues (*p* < 0.05). Our results were consistent with others indicating that oyster gills could be a better tissue for detecting HuNoVs in artificially contaminated oysters (Wang et al., 2008a; Suffredini et al., 2012).

Thirty-six oysters were collected randomly between March 2014 and February 2015 from retail market A and B in shanghai (Yu et al., 2016) and tested for both GI and GII HuNoVs by both assays. From June 2014 to August 2014, no HuNoV could be detected by both assays. The highest detection rate for HuNoV occurred in January followed by December (data not shown). Overall, ISC-RT-qPCR provided a better detection rate than that of RT-qPCR for both GI and GII HuNoVs. GII HuNoVs could by detected only in one oyster by ISC-RT-qPCR assay but not by RT-qPCR assay. The detection rates of GI HuNoVs in G, D, and O tissues were 33.3, 25.0, and 19.4% by ISC-RT-qPCR; and were 5.6, 11.1, and 11.1% by RT-qPCR, respectively. We further demonstrated that G tissue could be a perfect tissue for ISC-RT-qPCR method (Figure 2A) for GI HuNoV. Positive detection of GI HuNoVs in O or D tissues was always associated with positive detection of GI HuNoVs in G tissues measured by ISC-RT-qPCR. However, for RT-qPCR assay, it was difficult to determine whether the oyster was contaminated by GI HuNoVs if only G tissues were tested (Figure 2B) as O or D tissues could be positive when G tissues were negative.

Compared with the artificially inoculated oysters, a low detection rate (2.8%) and limited distribution site (only in G tissue) for GII HuNoVs was found in retail oysters (Table 4B). It is possible that the difference was due to the variations in sample collections, such as the samples were collected at different time and different locations. Oysters used for artificial inoculation were collected at a single time point and a single location and retail oysters were collected at two other locations over a period of a year. It is also possible that the titer of GII HuNoVs in retail oysters was much lower than that in artificially inoculated oysters. Our results were consistent with others who demonstrated that GII HuNoV was not predominant genotype in oyster-related outbreaks (Le Guyader et al., 2012; Yu et al., 2015) and GII HuNoV could be less concentrated in oysters and have less persistence in oyster tissues than GI HuNoV (Maalouf et al., 2011; Le Guyader et al., 2013; Yu et al., 2015).

Overall, ISC-RT-qPCR was more sensitive that RT-qPCR in clinical and oyster samples. More HuNoVs could be detected in retail oysters by ISC-RT-qPCR. The detection limit for both GI and GII HuNoVs were lower in clinical samples. The enhanced sensitivity of ISC-RT-qPCR might be due to the fact that the method effectively concentrates, sequesters HuNoVs away from complex oyster tissues or stools, and effecting a more-thorough removal of RT-PCR inhibitors. There are a couple of other advantages for ISC-RT-qPCR over RT-qPCR. Firstly, the ISC-RT-qPCR method avoids the RNA extraction step, which generally the most time-consuming procedure. It does not require a chemical extraction of viral RNA, nor the transfer of chemically-extracted viral RNA, nor the transfer of the released viral RNA from the immobilized magnetic beads, to a separate reaction container for amplification. Secondly, the ISC-RT-qPCR method only needs 30 min of incubation to allow viruses to bind to prior-coated binding/PCR reaction containers. It can significantly reduce sample processing time and costs. Thirdly, RT-PCR inhibitors can be easily removed in the course of ISC-RT-qPCR by three washing steps. Fourthly, ISC-RT-qPCR can be easily adapted for use in an automated system for multiple samples. More importantly, ISC-RT-qPCR assay provided a better estimate for infectivity of HuNoV. In contrast to RT-qPCR, only the genomic RNA from encapsulated viral RNA could be amplified. Therefore, ISC-RT-qPCR positive oyster samples have a higher possibility to have infectivity than RT-qPCR positive oysters. Overall, ISC-RT-qPCR method has the great potential for detecting HuNoVs rapidly and efficiently in clinical, environmental and food samples.

The detection of HuNoVs from food samples other than oysters has been challenging (Schwab et al., 2000; Sair et al., 2002; Rutjes et al., 2006). Most of contaminated food samples contained much less HuNoVs than oysters. Complicated processes are required to concentrate viruses, to release their viral genomes, and remove RT-PCR inhibitors from different food matrix. ISC-RT-qPCR method could simplify steps in virus concentration, viral extraction, removal of RT-PCR inhibitors with enhanced sensitivity than traditional RT-qPCR assay and have a great potential to use in food samples other than oysters. Currently, we are in the process of testing if this method could be used in detection of HuNoVs in produce and environmental samples.

## Author contributions

DW and ZZ designed the experiments. ZZ, DW, ZT, and QW carried out experiments. ZZ, DW, ZT, QL, and QW analyzed the data and experimental results. ZZ, DW, and PT wrote and modified the manuscript. XS provided laboratory equipment and place.

### Conflict of interest statement

The authors declare that the research was conducted in the absence of any commercial or financial relationships that could be construed as a potential conflict of interest.
